# Advantages of the Combined Use of Cyclodextrins and Chitosan in Drug Delivery: A Review

**DOI:** 10.3390/pharmaceutics18020156

**Published:** 2026-01-25

**Authors:** Paola A. Mura

**Affiliations:** Department of Chemistry Ugo Schiff (DICUS), University of Florence, Via Schiff 6, Sesto Fiorentino, 50019 Florence, Italy; paola.mura@unifi.it; Tel.: +39-055-4573673

**Keywords:** cyclodextrins, chitosan, chitosan-cyclodextrin combined use, enhanced stability, increased loading capacity, better controlled release, enhanced permeation, improved bioavailability, synergistic effects

## Abstract

Cyclodextrins and chitosan are biomaterials largely used as pharmaceutical excipients due to their biocompatibility, biodegradability, and low/absent toxicity, associated with a number of favorable properties. In particular, cyclodextrins complexation is mainly utilized to improve the physicochemical and biological properties of drugs, including solubility, stability, and bioavailability, and to reduce their irritating effect. Nevertheless, some disadvantages related to the fast removal of the complex from blood circulation after in vivo administration, and possible competition effects for interaction with cyclodextrin between the complexed drug and other molecules present in the biological environment, can reduce their efficacy as drug carriers. On the other hand, chitosan is widely employed to take advantage of its mucoadhesive, controlled/targeted release, and permeation-enhancing properties. However, its almost complete insolubility in water and poor affinity towards hydrophobic molecules (as most drugs are) are considered its main drawbacks, which could strongly limit its applicability. Due to the several beneficial properties of both cyclodextrins and chitosan, their joint use could provide additional favorable effects in drug delivery and help overcome their disadvantages, in particular by combining the complexing/solubilizing ability of the former towards hydrophobic molecules with the mucoadhesive and controlled/targeted release properties of the latter. The present review is intended to provide a critical and comprehensive summary of the main relevant investigations performed in the last twenty-five years regarding the applications and possible advantages that can be obtained by the combined use of cyclodextrins and chitosan in the development of more effective drug delivery systems.

## 1. Introduction

With the continuous advances in material chemistry research, the use of oligosaccharides and polysaccharides derived from natural sources in the pharmaceutical and biomedical fields has experienced a significant increase. In fact, over the last few decades there has been an exponential growth in the use of both cyclodextrins (a class of macrocyclic oligosaccharides obtained from enzymatic degradation of starch) and chitosan (a polysaccharide mainly obtained from the shells of crustaceans), as well as their derivatives, in pharmaceutical, biomedical, cosmetic, and environmental sectors, as confirmed by several reviews [1,2,3,4,5,6,7,8]. In particular, thanks to the biocompatibility, biodegradability, and absence/low toxicity of these materials, both the inclusion complexation ability of cyclodextrins (with all its consequent possible positive effects) and the mucoadhesive and controlled/targeted release properties of chitosan have been largely exploited in the development of pharmaceutical formulations intended for different administration routes, as well documented by numerous reviews [9,10,11,12,13,14,15].

However, in the case of cyclodextrins, the fast removal of the drug–cyclodextrin complex from the blood circulation after its in vivo administration, as well as possible competition effects in the biological environment between the complexed drug and other molecules for interaction with cyclodextrin, can reduce their actual efficacy as drug carriers.

On the other hand, in the case of chitosan, its almost complete insolubility in water, and its poor affinity towards hydrophobic molecules (as most drugs are), are considered its main drawbacks, which could strongly limit the possibility of adequately exploiting its several potential beneficial properties.

Some recent reviews illustrated and compared the possible pharmaceutical applications of cyclodextrins and chitosan and the related advantages and benefits that have been obtained [16,17,18,19].

Due to the number of beneficial properties of both cyclodextrins and chitosan, their joint use could provide additional favorable effects in drug delivery and help overcome their disadvantages, in particular by combining the complexing/solubilizing ability of the former towards hydrophobic molecules with the mucoadhesive and controlled/targeted release properties of the latter.

Nevertheless, despite this dual strategy having been successfully explored by several authors, to the best of our knowledge, to date there are no reviews on this topic.

Therefore, the present review is intended to provide, for the first time, a critical and comprehensive overview of the main relevant investigations performed over the last twenty-five years on the possible advantages, synergistic effects, and eventual limitations encountered in applying a strategy based on the combined use of cyclodextrins and chitosan for the development of new, more effective drug delivery systems.

## 2. Cyclodextrins

**Figure 1:**
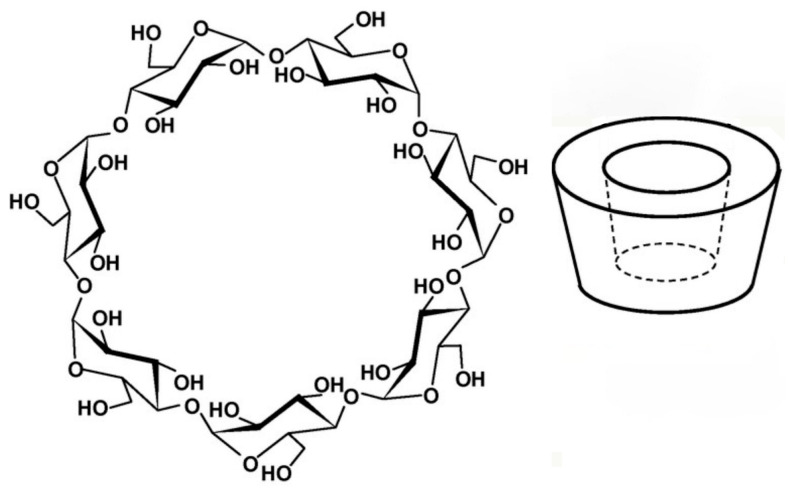
Schematic representation of βcyclodextrin.

Cyclodextrins (CDs) are a family of cyclic, amphipathic structures formed by (α-1,4)-linked D-glucopyranose units, produced by the enzymatic degradation of starch. They have a truncated-cone shape, where the secondary and primary hydroxyl groups are situated at the wider and narrower edges of the cavity, respectively (see Figure 1) [9].

The presence of the hydroxyl groups is responsible for the hydrophilic character of their outer surface, while the inner cavity, lined by hydrogen atoms and glycosidic oxygen bridges, has a somewhat hydrophobic character. This peculiar structure enables CDs to form host–guest type inclusion complexes in aqueous solutions by accommodating within their cavity a wide variety of lipophilic molecules of proper dimensions [9]. These complexes are characterized by the absence of covalent bonds between guest and host molecules, and the complexed molecules are in dynamic equilibrium with the free ones. Complexation with CDs allows the physicochemical and biological properties of the guest molecules to be suitably improved [20]. Due to their biocompatibility, biodegradability, absence/low toxicity, and relatively low cost, CDs have been widely used as excipients in medical, pharmaceutical, cosmetic, and food industries [11].

The natural CDs used in industrial-scale production, and the only ones utilized in the pharmaceutical sector, consist of six, seven, and eight glucose units and are called αCD, βCD, and γCD, respectively. Among these, βCD has the cavity with the most suitable dimensions to host several kinds of guest molecules; therefore, it is still the most used CD in the pharmaceutical field. However, natural CDs, and especially βCD, have limited water solubility due to the presence of intermolecular hydrogen bonds between secondary hydroxyl groups of adjacent glucose units, which can limit their applicability [20]. Therefore, different kinds of modified CDs have been developed to enhance solubility and complexing ability of the natural ones. The introduction of suitable substituents on the CD hydroxyl groups, such as hydroxypropyl groups (HPαCD, HPβCD, and HPγCD derivatives), or, in the case of βCD, methyl (MβCD), sulfobutylether (SBEβCD), or carboxymethyl (CMβCD) groups, has allowed a considerable improvement in water solubility [20].

Monographs for natural CDs are reported in the European Pharmacopoeia (Ph. Eur.), the United States Pharmacopeia/National Formulary (USP/NF), and the Japanese Pharmaceutical Codex (JPC). Moreover, Ph. Eur. has a monograph for HPβCD, while USP/NF has monographs for HPβCD and SBEβCD. Because of their very low oral bioavailability, all native CDs, as well as most of their derivatives, are not toxic after oral administration. Regarding parenteral use, only γCD among the native CDs is considered suitable for intravenous administration, presenting the best toxicological profile, while αCD and βCD, due their lower solubility, can give renal toxicity [21]. Among the CD derivatives, only HPβCD and SBEβCD have been approved for parenteral use, while renal toxicity has been reported for methylated-βCD derivatives; however, the randomly methylated-βCD (RMβCD) can be used in topical or nasal formulations [22]. Furthermore, all natural CDs have been included in the “GRAS” (Generally Recognized as Safe) list of the United States FDA (Food and Drug Administration) and can be used as food additives. Finally, all natural CDs, as well as HPβCD, SBEβCD, and HPγCD are present in the FDA’s list of Inactive Pharmaceutical Ingredients.

The complexing ability of CDs has proven to be a valuable tool for improving the physicochemical properties of drugs, increasing their solubility and bioavailability, enhancing stability against oxidation, hydrolysis, and light degradation, reducing the evaporation of volatile substances, modulating their release, limiting irritating effects against mucous membranes, tissues, or skin, and masking bad smelling or bitter taste. Finally, they can also be used to convert liquid drugs into powders and to avoid incompatibility reactions between components in formulations [23].

By virtue of their numerous profitable effects associated with absent/low toxicity and biocompatibility, CDs have found useful applications in several kinds of drug delivery systems designed for different administration routes, ranging from the conventional oral route [24], to the topical [25] transdermal [26], nasal [27], ocular [28], and parenteral [29] ones, as well as carriers in gene therapy [30].

However, despite the numerous advantages that can be obtained by drug–CD complexation, there are some important factors that can limit CD applicability and need to be considered. In particular, to be suitable for CD complexation, drugs must have adequate lipophilicity and proper dimensions in order to have good affinity for the hydrophobic CD cavity and to be able to fit inside it, at least in part.

Moreover, considering that CDs have rather large molecular weights (972, 1132, and 1297 for αCD, βCD, and γCD, respectively, and even larger for CD derivatives), the single drug dose should be low enough not to cause formulation problems [9].

Finally, for each potential guest molecule to be complexed, careful selection of the most suitable CD should be made, since adequate values of the complex stability constant are necessary depending on the goal to be achieved through the drug–CD complexation.

## 3. Chitosan

**Figure 2:**
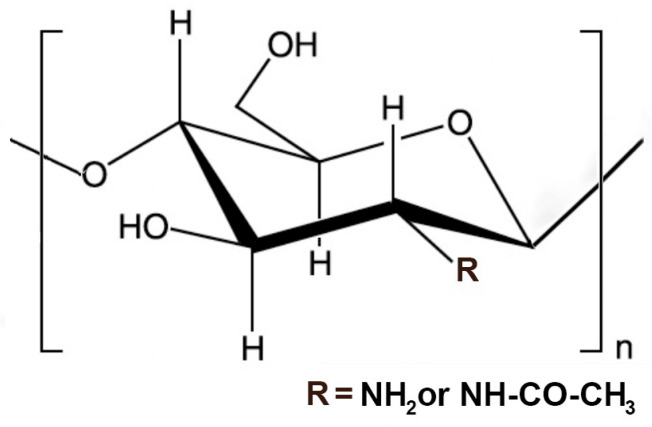
Representative structure of chitosan.

Chitosan (CS) is a linear polysaccharide produced via partial deacetylation, by alkaline hydrolysis, of chitin, the most plentiful biopolymer in nature after cellulose [31]. CS is a co-polymer comprising randomly distributed N-acetyl-D-glucosamine and deacetylated D-glucosamine (D-units) units connected by β-1,4 glycosidic bonds (Figure 2).

CS is practically insoluble in water; however, it is soluble in acidic solutions due to the protonation of its amino groups. The positive charge is considered responsible for important properties of CS, including its capability to interact with a variety of molecules, including the negatively charged constituents of mucosal surfaces and cell membranes [32], and to form polyelectrolyte complexes with different kinds of polyanionic compounds (PECs) [33].

CS is endowed with numerous important biological properties, such as antifungal, antibacterial, anti-viral, antitumor, antioxidant, and anti-inflammatory activities [13]. These properties, together with other important characteristics, including non-toxicity, biocompatibility, biodegradability, mucoadhesiveness, permeation-enhancing power, and last but not least, low cost and wide availability on the market, have led to its extensive application not only in a variety of drug delivery systems [15], but also in gene therapy [34] and tissue engineering [35]. Furthermore, its easy derivatization also contributes to further improving its physicochemical properties and stability and expanding its applications [36].

The exceptional combination of favorable biological and physicochemical characteristics of CS, as described above, together with its safety in use, being recognized by the FDA as a GRAS (Generally Recognized as Safe) material, as well as its great technological versatility, since it is able to form films, micro- and nanoparticles, chemical and physical hydrogels, including stimuli-responsive “in situ” gelling systems, has made this polymer an essential tool in the pharmaceutical formulation field. In fact, its advantageous properties have been exploited in the development of a variety of drug delivery systems intended for oral [37], topical [38], transdermal [39], buccal [40], ocular [41], nasal [42] rectal [43], and vaginal [44] application.

However, regardless of the number of advantageous properties and the wide range of applications provided, the use of CS in the pharmaceutical field still presents some critical issues.

The extremely low water solubility of CS is regarded as the major disadvantage for its use in various sectors of drug delivery. In fact, CS is soluble, and can thus exert its mucoadhesive ability, only at acidic pH (pH < 6), by virtue of the ionization of its amino groups. Fortunately, both these issues, i.e., poor mucoadhesiveness and almost complete insolubility at pH values close to neutrality, can be solved by using CS salts [45] or by choosing appropriate derivatives endowed with enhanced water solubility, mucoadhesiveness, and mechanical strength [46].

However, it must be considered that the increasingly numerous CS derivatives, obtained by virtue of its ease of chemical modifications, require a careful characterization of their physicochemical and biopharmaceutical properties, as well as an adequate assessment of their possible cytotoxicity, in order to establish their proper and safe use [47]. Furthermore, all CS derivatives necessarily have a higher cost than the native polymer.

Moreover, despite its large availability in nature, CS production is not a minor concern. In fact, strict control is necessary to elude problems of uncontrollable hydrolysis or undesired chemical modifications that could happen during polymer isolation [48]. Additionally, due to the high quality necessary for products designed for biomedical and pharmaceutical use, several purification processes are also needed for unmodified CS before it can be used [49].

Finally, another critical issue is the extremely wide choice of CS types available on the market, varying in molecular weight and acetylation degree and pattern (i.e., the distribution of comonomers sequences), which can markedly affect its properties and therefore needs adequate characterization to avoid risks of non-reproducible or unexpected results [47,50].

## 4. Rationale for the Combined Use of Cyclodextrins (CDs) and Chitosan (CS)

Considering the numerous and different beneficial effects offered by CDs and CS, several researchers considered it interesting to investigate the eventual further advantages that could be obtained in the drug delivery field by their joint use, due to combinations of their properties and/or possible synergistic effects.

Two main types of approaches have been followed in this regard (see following Section 6 and Section 7). The first one, which is the simplest and most widely applied, consists of directly exploiting the benefits deriving from the co-presence of CD and CS, which are added separately to the pharmaceutical formulation. This strategy also includes the possibility of using negatively charged CDs, which can not only act as complexing/solubilizing agents towards the drug, but also interact with the positively charged CS.

The second approach consists instead of the development of suitable “biocomposites” or “conjugates” by grafting CDs onto CS or by chemically crosslinking them, so as to obtain new bifunctional materials combining the favorable properties of both components. Clearly, adequate studies are necessary in this case to evaluate the actual biocompatibility, biodegradability, and absence of cytotoxicity of the new compounds.

**Figure 3:**
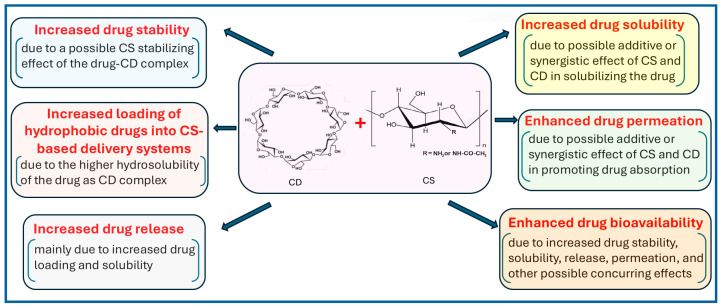
Schematic drawing of the possible advantages expected in drug delivery by the combined use of cyclodextrin (CD) and chitosan (CS).

The main possible advantages expected from these two kinds of approaches are the same and are illustrated in the following scheme (Figure 3).

## 5. Preliminary Investigations on the Effects on the Separate or Combined Use of CD and CS on Drug Solubility and Permeability

Owing to the several favorable properties of both CD and CS, the possible further benefits actually obtainable by their combined use in terms of increased drug solubility and/or permeability, with respect to their separate use, have been investigated in a series of preliminary studies.

For example, the influence of βCD or HPβCD and CS, when used jointly or separately, on the solubility and permeability through Caco-2 cells of glyburide, an oral hypoglycemic drug with variable bioavailability mainly due to its very low water solubility, has been examined [51]. The authors found that βCD, and even more so HPβCD, were more effective than CS in improving drug solubility, and that the simultaneous presence of CD and CS gave rise to a four to five times reduction in the CD solubilizing action, attributed to possible competition between CS and the drug for interaction with the CD. On the other hand, CS showed a higher permeation-enhancing power than CDs; however, in this case, a synergistic positive effect was observed as a consequence of their concomitant presence. This last finding has been attributed by the authors to the reduction by CS of the drug–CD complex stability, as described above; in fact, such an effect increased the amount of free drug in solution available for absorption, allowing the CD permeation-enhancing ability to be better brought out [51].

The influence of CS presence on the complexing and solubilizing power of βCD or HPβCD towards a series of selected acidic or basic drugs with different water solubility and lipophilicity was investigated by phase solubility studies [52]. The authors found that the addition of CS to the CD complexation medium gave rise to a reduction in the CD complexing ability towards all the selected drugs, regardless of their different characteristics. This result was ascribed to possible CD-CS interactions that hindered drug-CD complex formation and thus caused a reduction in the stability constant. Conversely, for the influence of CS on CD solubilizing efficiency, favorable, negative, or non-significant effects were revealed, depending on the kind of drug considered. This outcome indicated that the final solubilizing effect towards the drug was instead affected by the formation of drug-CS and/or CS-(drug-CD complex) interactions, which were different from drug to drug [52].

Maestrelli et al. [53] examined the effect of the joined utilization of randomly- methylated-βCD (RMβCD), CS, and bile components (dehydrocholic or ursodeoxycholic acids and their sodium salts) on the solubility and permeability through Caco-2 cells of oxaprozin, a non-steroidal anti-inflammatory agent with very low water solubility. Ternary coground drug-RMβCD-CS systems exhibited a clearly greater dissolution rate than binary drug-RMβCD systems, and a further increase was obtained by adding the bile components. Moreover, CS addition enhanced drug permeability compared to drug-RMβCD systems, and a further increase was obtained in the presence of the sodium salt of dehydrocholic acid [53].

Based on these studies, it can be concluded that, due to the complexity of the concomitant and sometimes competitive interactions between drug/CS, drug/CD, (drug-CD)/CS, and CD/CS, it is not possible to foresee a priori the effect of CS presence on the CD complexing/solubilizing ability towards a given drug, as well as on drug permeability, and it has to be determined on a case-by-case basis.

## 6. Delivery Systems Based on the Concomitant Use of CD and CS, Separately Added to the Formulation

**Figure 4:**
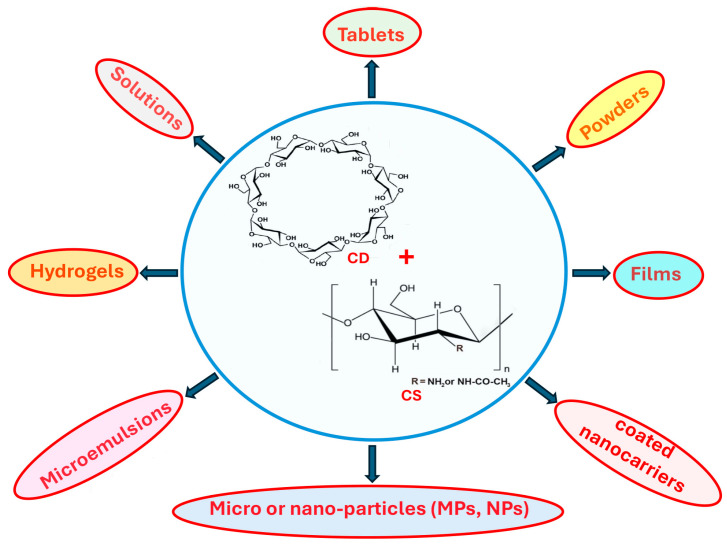
Schematic drawing of the different kinds of dosage forms or drug delivery systems exploiting the combined use of cyclodextrin (CD) and chitosan (CS), including tablets ([53,56–58]), powders ([97]), films ([76–84]), coated nanocarriers ([87]), micro or nanoparticles ([104–149]), microemulsions ([93]), hydrogels ([68,95,96]), solutions ([94]).

This is the simplest and most versatile strategy, since it allows the direct use of CDs and CS, both as native or derivative products, without requiring any previous chemical modification or the addition of any particular reagent. Therefore, as illustrated in Figure 4, this approach can be easily applied to any type of drug dosage form or delivery system designed for different administration routes.

### 6.1. Solid Oral Delivery Systems Containing CDs and CS

The oral route is still the most widely utilized administration way, and solid dosage forms are the most commonly used ones, due to their several positive aspects, including easy and pain-free self-administration, high patient compliance, safety in use, high stability, low production cost, and wide formulation versatility. Nevertheless, several drugs present limited/variable oral bioavailability, mainly related to poor solubility and/or limited permeability, or lack of stability in the gastrointestinal tract, owing to degradation problems at gastric pH or by the various digestive enzymes present.

The combined use of CD and CS can be fruitful in overcoming most of the above issues by exploiting possible synergisms in their solubilizing, stabilizing, permeation-enhancing, and site-specific targeting abilities.

In this regard, some authors have taken advantage of the site-specific degradation of CS by colon bacteria, as well as of the solubilizing effect of CDs, to develop effective colon-targeted delivery systems, even though they required an enteric coating to prevent premature drug release in gastric medium (pH 1.2), due to the solubility of CS in acidic solutions. For example, Maestrelli et al. [54] developed Eudragit-coated CS-based microspheres loaded with ketoprofen as a complex with HPβCD, aimed at colon-targeted delivery. The system exploits CD complexation to improve drug solubility and vectorization in CS microspheres to benefit from the enzyme-specific biodegradability and mucoadhesive properties of this polymer. An about two times higher (*p* < 0.001) permeation rate of ketoprofen through Caco2-cells was found for CS microspheres loaded with the complexed drug compared to those containing the free drug, evidencing a synergistic effect between CD and CS in enhancing drug absorption [54]. This effect has been previously reported [51,55] and has been attributed to the proven capacity of CDs to reversibly extract phospholipids from biological membranes, thus promoting tight junction opening by CS [55], as well as to a decrease in the stability of the drug-CD complex in the presence of CS [52], which makes a greater amount of free drug molecules available for permeation.

A Quality-by-Design (QbD) approach has been applied to develop CS-Ca alginate microspheres designed for celecoxib colon delivery, suitable for both local (carcinogenesis prophylaxis) and systemic (arthritis chronotherapeutic treatment) therapy [56]. The system takes advantage, on the one hand, of drug ternary complexation with HPβCD and PVP to improve its solubility and, on the other hand, of the mucoadhesive properties and colon-specific biodegradability of CS. The QbD strategy allowed the optimization of the formulations in terms of entrapment efficiency and drug amount released after a given time in the colon environment for both local and systemic uses [56].

Mucoadhesive buccal tablets have been developed aimed at prolonged local or systemic release of clonazepam [57]. Among the different polymers tested for swelling, erosion, and in situ residence time, the 30/70 poloxamer/CS mixture gave the best values. Kollicoat was instead used as a backing layer, providing unidirectional drug delivery from systemic release buccal tablets. Randomly methylated-βCD (RMβCD) was selected as the best CD to increase drug solubility, allowing a 100% increase in drug release from local delivery tablets. Moreover, in vitro permeation studies from systemic release tablets showed that drug loading as an RMβCD complex allowed a fivefold increase in drug flux and permeability compared to tablets loaded with the plain drug [57].

A completely different effect was obtained by Anraku et al. [58], who exploited the spontaneous formation of a poorly soluble interpolymer complex between cationic CS and anionic SBEβCD to realize slow-release tablets of famotidine, a potent H2 receptor antagonist [57]. The authors observed that, after exposure to the dissolution medium, gelation happened on the surface of the CS-SBEβCD tablets, forming a sort of barrier to famotidine diffusion, thus slowing its release over 24 h (2 h at pH 1.2 and 22 h at pH 6.8). In contrast, 100% drug release after only 2 h at pH 1.2 was instead achieved from CS-lactose tablets used as a reference [58].

### 6.2. CS-Based Hydrogels Containing CDs

The capacity of CS to form hydrogels has been widely utilized to develop a variety of drug delivery systems for both local and systemic controlled release [59,60,61]. Chemical hydrogels are prepared by CS crosslinking via covalent bonds with other polymers [62,63]. Physical reversible hydrogels are instead formed by spontaneous electrostatic interactions between positively charged CS and various negatively charged polymers. These latter systems are generally preferable, since they do not require the use of crosslinking agents that could be potentially toxic; moreover, the absence of CS chemical modifications allows maintenance of all its beneficial characteristics, including biocompatibility and biodegradability [61]. However, physical hydrogels present a drawback in terms of less control over their degradation/dissolution process and, consequently, drug release behavior [60]. On the other hand, the formation of polyelectrolyte complexes (PECs) can be helpful in properly modulating the drug release from CS physical hydrogels [64].

Interestingly, stimuli-responsive in situ gelling systems can also be obtained, i.e., liquid formulations able to undergo to a rapid transformation into a gel, mainly activated by physiological pH or temperature variations [65]. In situ gelling systems have the advantage of enabling easy application of the liquid formulation at the administration site, combined with a fast in situ gelation, resulting in a strong viscous system that facilitates polymer mucoadhesive action and extends local residence time [66].

However, some drawbacks still restrict the use of hydrogels in the development of drug delivery systems, particularly their limited loading efficiency towards poorly water-soluble drugs, which are not prone to interact with hydrophilic networks. Therefore, their performance, including that of CS hydrogels, could be further improved by profiting from the favorable complexing/solubilizing properties of CDs, especially in the case of hydrophobic drugs. The positive effect of CD presence has already been proven in the case of polyethyleneglycol-based hydrogels [67].

A particular type of CS-based pH-responsive hydrogel has been prepared based on electrostatic interactions between negatively charged carboxymethylβCD (CMβCD) and CS [68]. Self-assembled microparticles were then obtained by dispersion of the lyophilized hydrogel in a phosphate-buffered solution supplemented with EDTA-functionalized CS. the microparticles, loaded with bovine serum albumin (BSA) as a model protein, exhibited a pH-sensitive controlled release of the protein for up to 40 h [68].

A sponge-like material was prepared starting from physical hydrogels obtained by acidification of an aqueous suspension of co-milled powders of CS and a water-soluble anionic polymeric βCD (able to interact with each other via polyelectrolyte complex formation), and subsequent freeze-drying [69]. The rheological and swelling properties of the obtained sponges depended on their CS: polymeric βCD *w*/*w* ratios. The sponge with a 3:3 *w*/*w* ratio was selected for loading with the poorly soluble model drug chlorhexidine and showed activity against *Streptococcus aureus* for up to 30 days in agar diffusion tests [69].

### 6.3. CS-Based Films Containing CDs

The high density of amino and hydroxyl groups in the CS backbone is responsible for its film-forming capacity and its ability to interact with other substances. The final film properties are strongly dependent on different factors, including the type of acid used to dissolve CS, the polymer molecular weight (the higher the molecular weight, the greater the film resistance and barrier properties), the deacetylation degree (the lower the deacetylation degree, the lower the water vapor permeability and the higher the tensile strength), and the presence of additives such as plasticizers or other polymers [70].

Films of CS and its derivatives are widely used in biomedical, pharmaceutical, cosmetic, and packaging applications due to their biocompatibility, biodegradability, and antioxidant and antimicrobial activities. In particular, regarding the pharmaceutical field, CS films have found applications in wound care, as wound dressings, and in a variety of drug delivery systems designed for different administration routes, exploiting their mucoadhesive properties to obtain a prolonged in situ residence time and controlled and sustained drug release [71].

In order to improve the mechanical, physicochemical, and biopharmaceutical properties of CS films and further extend their applications, it has often been used in mixtures with various other polymers, both synthetic, such as PVP [72], or semisynthetic, such as methyl-, hydroxypropylmethyl-, and hydroxyethyl-cellulose [73], or natural ones, such as alginate, pectins, or xanthan [74,75].

Some authors have proved that an additional way that can be utilized for improving the performance of CS films, particularly in terms of drug loading capacity, drug release behavior, and drug stability, is the addition of CDs.

For instance, a mucoadhesive film for buccal administration of the poorly soluble anti-inflammatory drug flufenamic acid was developed using CS and KollicoatIR as mucoadhesive and film-forming polymers, respectively, glycerol as plasticizer, and loading the drug either as such or as a complex with HPβCD [76]. Phase solubility studies showed a synergism between CS and CD in improving drug solubility, with a greater than simply additive effect compared with the respective drug-CS and drug-HPβCD binary systems, and a 2.6-fold increase in the CD complex stability constant in the presence of 2.0% CS. To explain this result, the authors hypothesized that CS contributed to enhancing CD complexation ability towards the drug by establishing intermolecular interactions like hydrogen bonds and/or Van der Waals forces. The improved drug solubility and dissolution enabled prolonged and complete release over 4–5 h, while the film containing the free drug exhibited slower and incomplete release [76].

The effect of using native βCD or soluble polymeric βCD (epichlorohydrin-β-CD, EPIβCD) to increase the effectiveness of CS bucco-adhesive films loaded with bupivacaine.HCl or triclosan as poorly water-soluble model drugs has been investigated [77]. The authors showed that the type of CD used can differently influence film properties and drug release due to their different affinities for interactions with CS and the drugs. In fact, despite the greater solubilizing power of EPIβCD compared to βCD towards both drugs, their effect on drug release rate differed depending on their different interactions with CS; namely, βCD was the best partner for bupivacaine films, in terms of swelling behavior, mucoadhesion, and drug release, while EPIβCD was the best for triclosan films, enabling the highest drug release rate [77].

Mucoadhesive CS-based films have been developed to increase residence time at the application site and prolong ocular delivery of cyclosporin in the treatment of dry eye disease [78]. HPβCD was added to increase drug solubility and concentration at the ocular surface and then, possibly, its absorption by passive diffusion. However, release studies unexpectedly showed that the presence of HPβCD did not significantly increase drug release, probably because its solubilizing effect was not enough to compete with the slow swelling behavior of the films [78]

CS films loaded with different concentrations of antimicrobial essential oils complexed with βCD were realized and evaluated for antimicrobial, mechanical, and physical properties [79]. The use of essential oils as βCD complexes enhanced the antimicrobial action of CS films against *Escherichia coli*, *Salmonella enterica,* and *Streptococcus aureus,* probably due to their higher dispersibility in water compared to free oils, enabling improved contact with pathogens. Moreover, the presence of the βCD complex increased film tensile strength and significantly reduced water vapor permeability, thereby increasing barrier properties, probably in virtue of the formation of hydrogen bonds with CS [79].

Analogously, Bai et al. [80] prepared CS films loaded with antimicrobial/antioxidant natural essential oils encapsulated in soluble polymeric βCD (EPIβCD). Incorporation of essential oils as CD complexes allowed high loading efficiency (reaching 74 and 79% for cinnamaldehyde and thymol, respectively), dramatically increasing the antimicrobial/antioxidant activity of the CS film. Moreover, the presence of EPIβCD improved the film thermal stability and increased water solubility and swelling degree thanks to the good hydrophilicity of the complexed oils and interactions among CS, the plasticizer glycerol, and the CD complexes [80].

In a similar way, incorporation of HPβCD into CS films was exploited to improve their loading ability for the volatile, hydrophobic antimicrobial agent carvacrol [81]. The use of CD together with glycerol added as a plasticizer and suitable moisture conditions allowed an increase of more than 200 times in the % of carvacrol retained in CS films, which showed antimicrobial action against *Escherichia coli* and *Streptococcus aureus*. No antimicrobial activity was found for CS films without CD, in which, irrespective of the amount of glycerol and water added, the amount of loaded carvacrol was always lower than 1% and did not reach the minimum inhibitory dose [81].

Zarandona et al. [82] designed CS films containing the bioactive ingredient 2-phenylethanol as a βCD inclusion complex. The authors proved that the presence of CD improved mechanical properties of CS films by increasing tensile strength and enhanced stability of the bioactive component, markedly reducing its evaporation and allowing 90% retention vs. 8% retention obtained in the absence of βCD [82].

The potential of adding soluble polymeric βCD (EPIβCD) to CS films to improve loading and better control release of carbendazim, used as a plant healthcare material against *Sclerotinia sclerotiorum*, has been investigated [83]. Formation of 1:1 mol:mol host/guest complexes was demonstrated, resulting in an approximately 18-fold increase in drug solubility and enabling controlled release, thereby prolonging drug duration of action, as proved by in vivo experiments [83].

Jiang et al. [84] developed a sustained-release CS film containing rutin as a βCD complex and glucoamylase. Complexation of rutin with βCD allowed its successful loading into the CS film, thus strongly enhancing antioxidant activity; on the other hand, the presence of the enzyme increased the release rate of rutin by destabilizing the complex via the hydrolyzation of βCD. Moreover, the presence of βCD improved film tensile strength and lowered water vapor permeability [84].

#### CS Films for Coating of Colloidal Carriers Containing CDs

CS films can also be exploited as coating materials, not only for conventional solid dosage forms but also for various colloidal carriers, like liposomes, niosomes, or nanoparticles, offering a fruitful way for improving their stability by conferring a positive charge to their surface, promoting mucoadhesion, and enhancing cellular uptake. Moreover, the CS surface can be chemically modified to attach targeting molecules to specific cells or tissues, and the positive surface charge facilitates interactions with negatively charged biological membranes [85].

On the other hand, CD complexation has been successfully exploited to enhance the performance of various nanocarriers, particularly in terms of enhanced entrapment efficiency of poorly soluble drugs, increased stability, and better control of drug release, resulting in enhanced drug therapeutic efficacy [86].

Further benefits could be obtained by the combined use of CS coating of nanocarriers and drug complexation with CDs.

In a recent study, the effect of combining drug-CD complexation, incorporation of the complex into niosomes, and CS coating of niosomes to improve curcumin delivery in the treatment of osteoarthritis was evaluated [87]. HPβCD was used as complexing agent to improve the solubility and stability of curcumin, and the obtained complex was loaded into niosomal vesicles aimed at controlling drug release, which were finally coated with CS to enhance their stability and further control drug release. To better evaluate the role of both HPβCD and CS in the performance of the final formulations, uncoated and CS-coated niosomes loaded with either the drug-HPβCD complex or the free drug were prepared and compared. Drug loading as a CD complex rather than as the plain drug allowed enhanced and more sustained release, and CS coating even further took part in this result while also enhancing the stability of the niosomal formulation. In vivo studies in rats pointed out a significantly (*p* < 0.05) more intense pain-relieving effect and longer duration of action for the CS-coated niosomal formulation loaded with the curcumin-HPβCD complex and also evidenced an additional anti-inflammatory effect of CS [87].

### 6.4. Nasal Formulations for Systemic or Nose-to Brain Drug Delivery Based on the Simultaneous Presence of CS and CD

The nasal route appears as an attractive and efficient way for administration of drugs for both local and systemic delivery due to several advantages, including the highly vascularized nasal mucosa, avoidance of hepatic first-pass metabolism, rapidity of action, non-invasiveness, painlessness, and good patient compliance [88]. Furthermore, the nasal route can allow direct drug delivery to the brain through the olfactory and trigeminal nerve pathways, bypassing the Blood–Brain Barrier (BBB) [89,90].

On the other hand, the presence of fast muco-ciliary clearance phenomena, mucus, and epithelial barriers, as well as the small administrable volume, can represent important limitations to effective drug absorption. Therefore, numerous strategies have been investigated to enhance the efficiency of nasal drug delivery, aimed at defeating the principal factors limiting effective absorption, such as the brief residence time at the administration site and poor permeation through the epithelial membrane, mainly by the use of mucoadhesive polymers and adsorption enhancers [91].

The biocompatibility, biodegradability, and safety in use of CS, together with its mucoadhesive and penetration-enhancing properties, make this polymer an excellent candidate for the development of nasal drug delivery systems, helping to overcome the described drawbacks [42].

CDs have also been recognized as powerful excipients in nasal drug delivery, particularly by virtue of their solubilizing ability and absorption-enhancing effect [27,92]. Consequently, the combined use of CS and CDs should provide further benefits in improving the performance of nasal drug delivery systems.

For instance, mucoadhesive microemulsions (MEs) have been developed for intranasal administration of buspirone hydrochloride (a selective anxiolytic drug), aimed to improve its bioavailability and achieve high brain levels [93]. With this purpose, the authors evaluated the effect of the addition of CS aspartate, a hydro-soluble CS salt with mucoadhesive properties, alone or in combination with HPβCD, considering the known ability of CDs to act as absorption enhancers in nasal delivery [27,92]. MEs containing CS or CS and HPβCD allowed 1.3- and 1.7-fold increases, respectively, in mucoadhesive power. Ex vivo permeation studies through excised sheep nasal tissue showed that the cumulative amount of drug permeated after 6 h reached 75.5% and 100%, respectively, with respect to the 65% achieved for the starting ME formulation. Moreover, intranasal administration to rats resulted in rise in C_max_ values in brain that were 2.5 (starting ME), 3.7 (ME with CS), and 4.3 times (ME with CS and HPβCD) higher than the intravenous solution, proving a direct nose-to-brain transport of buspirone and very high brain-targeting efficiency. The best effectiveness of the MEs holding CS and HPβCD was ascribed to the mucoadhesive power of CS, which prolonged the ME contact time with the mucosa and promoted tight junction opening, together with the permeation-enhancing effect of CDs [93].

The potential of intranasal administration of carnosic acid to increase the levels of neurotrophins NGF (nerve-growth-factor) and BDNF (brain-derived neurotrophic factor) in the brain was evaluated [94]. With this aim, the authors evaluated the solubilizing effect of HPβCD on the drug, as well as the permeation-enhancing ability of CS. The very low solubility of the drug in Krebs–Ringer bicarbonate buffer (3 µg/mL) increased by about 900-fold in the presence of 0.143 M HPβCD. Ex vivo permeation studies through excised bovine olfactory mucosa showed that the presence of CS at 0.25% allowed an about eight times increase in drug permeation, attributed to the transient opening of the tight junctions, while no further increase was found when the CS concentration was increased to 0.5%. In contrast, intranasal administration of the drug-HPβCD solution in rats enabled a 50% increase in BDNF levels in the hippocampus region compared to controls but did not enhance NGF levels. On the contrary, intranasal administration of the drug-HPβCD solution containing 0.25% CS allowed twofold and threefold increases in BDNF and NGF levels, which was about 1.5–2.0-fold more than those achieved by parenteral administration [94].

A thermosensitive, mucoadhesive in situ hydrogel was realized for intranasal delivery of clonazepam, designed to obtain extended residence time at the administration site and controlled drug release, thus overcoming issues related to oral or parenteral administration [95]. The gel was based on poloxamer as a thermosensitive polymer and CS glutamate and sodium hyaluronate as permeation enhancers and mucoadhesive polymers, while randomly methylated-βCD (RMβCD) was utilized to increase the drug solubility. After preliminary screening to find the most suitable proportions between the excipients to obtain the desired gelation time, a series of gels at various concentrations of the clonazepam-RMβCD complex were prepared and suitably characterized, in comparison with analogous gels containing the plain drug. In vitro release studies, permeation experiments through Caco2-cells, and cytotoxicity studies pointed out the different roles of RMβCD, which not only almost doubled the drug release rate and significantly (*p* < 0.05) enhanced its permeation but also decreased drug cytotoxicity [95].

Another thermosensitive CS-based hydrogel, intended for nasal delivery of dimethyl fumarate and designed to avoid gastrointestinal side effects, has recently been developed [96]. The drug was added to the CS-glycerophosphate hydrogel precursor solution as a complex with HPβCD to increase water solubility, thereby enabling achievement of a high drug concentration in the reduced dose volume (max 200 µL) usually administered with nasal dispensers. A high loading efficiency was actually reached (around 92%) and remained stable for 21 days. The obtained loaded solution was able to form a hydrogel at nasal mucosa temperature (32–35 °C) in about 1–2 min, a time considered suitable to enable easy administration as a sol followed by rapid gelation, providing adequate permanence at the administration site [96].

Complexation with RMβCD was successfully exploited to improve both aqueous solubility and stability of dimethyl fumarate, used as an immunomodulator and cyto-protector towards neurons and glial cells, and the obtained complex was used to develop a CS-based mucoadhesive nasal powder designed for nose-to brain administration [97]. The powder, prepared by lyophilization, showed good mucoadhesive properties, providing a long in situ residence time, and rapid hydration, thus facilitating complex solubilization and, possibly, its absorption through the nasal mucosa [97].

### 6.5. CS-Based Microparticles (MPs) and Nanoparticles (NPs) Containing CDs

Particulate delivery systems such as microparticles (MPs, 1–1000 µm) and nanoparticles (NPs, 1–100 nm) have attracted growing interest due to the number of advantages they can offer by virtue of their peculiar mechanical, physical, and chemical properties deriving from their high surface-area-to-volume ratio. They have been revealed to be effective carriers for a wide range of applications, including injectable, nasal, and ocular delivery [98]. In particular, in recent years the utilization of NPs has exponentially increased, since their higher surface area improves their effectiveness as transport agents for drug delivery, including intravenous administration and intracellular delivery, and enhances their property to be functionalized for gene or tumor targeting [99].

The materials used to prepare MPs and NPs for medical applications need to have specific properties, such as biodegradability, biocompatibility, low/absent toxicity, and adequate hydrophilicity. CS, besides having all these properties, also has several additional beneficial characteristics, including, in particular, mucoadhesiveness and permeation-enhancing power. For these reasons, CS MPs and especially CS NPs have found wide applications as drug delivery systems for oral, ocular, nasal, pulmonary, and vaginal administration, as well as in tissue engineering and gene and anticancer therapy [100,101,102,103]. Moreover, the main preparation methods of CS MPs and NPs, i.e., ionic gelation or emulsion-based methods, generally involve simple procedures and require no or less use of organic solvents [100].

However, the principal drawback for a fruitful utilization of CS MPs and NPs is their low entrapment efficiency towards the large number of poorly water-soluble drug molecules, owing to the hydrophilic properties of CS and the aqueous environment generally used for CS MP and NP preparation.

Therefore, the development of new nanoparticulate carriers joining the advantageous properties of CS MPs and NPs with those of CDs, with particular reference to their solubilizing abilities, is expected to be successful in improving the bioavailability of drugs, particularly those belonging to classes 2 and 4 according to the BCS (Biopharmaceutical Classification System).

In fact, among the different formulation approaches based on the combined use of CS-CD, the one consisting of the development of “drug-in CD-in CS MPs or NPs” systems has been the most widely investigated and applied to different administration routes, including the nasal one intended for systemic or nose-to brain delivery.

**Figure 5:**
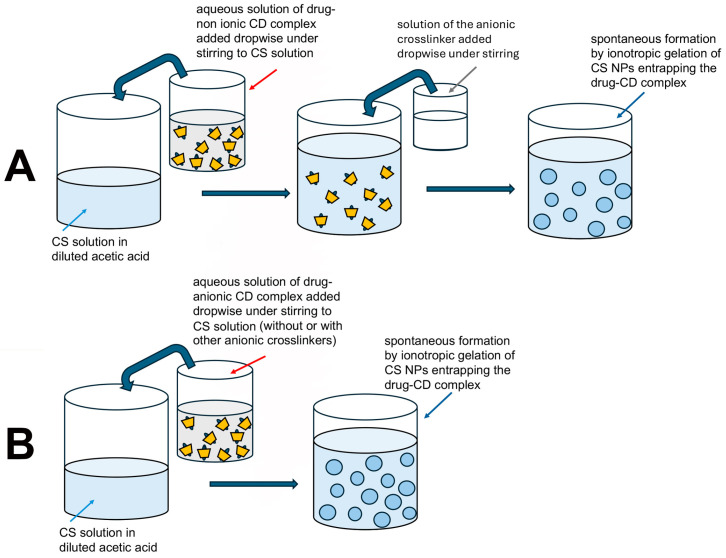
Schematic representation of the preparation of drug-in CD-in CS nanoparticles by the ionotropic gelation method using non-ionic CDs (**A**) or anionic CDs (**B**).

These “drug-in CD-in CS MPs or NPs” systems can be divided in two typologies, depending on whether they contain non-ionic or anionic CDs, owing to their different preparation conditions, as shown in the following scheme (Figure 5).

In fact, as can be seen in Figure 5, the preparation of “drug-in non-ionic CD-in CS MPs/NPs” by the ionotropic gelation method necessarily requires the addition of an anionic crosslinker to interact with the positively charged CS; on the contrary, this is not compulsory in the case of “drug-in anionic CD-in CS MPs/NPs”, where the anionic CD can act as a crosslinker.

To the best of our knowledge, no comparative studies have been conducted to date on the effectiveness of these two types of “drug-in CD-in CS MPs/NPs” as drug delivery systems. However, based on the data reported in the literature (that will be illustrated in detail in the following two paragraphs), both systems appear substantially analogous in terms of both successful performance and variety of applications across different administration routes.

#### 6.5.1. Drug-in Non-Ionic CD-in CS MPs and NPs

This strategy was successfully applied for the first time by Maestrelli et al. [104], who prepared CS NPs incorporating two hydrophobic model drugs (triclosan and furosemide) by ionotropic gelation of CS with sodium tripolyphosphate (TPP) in aqueous solutions in the presence of different concentrations of HPβCD. Complexation with HPβCD increased the solubility of both drugs, thus facilitating their entrapment into CS NPs and leading to an increase up to 4- or 10-fold (for triclosan and furosemide, respectively) in drug loading. However, phase solubility studies evidenced, in both cases, a clear decrease in the complex stability constant [104]. This result, as confirmed in other subsequent studies [52], was attributed to the presence of interactions between CS and CD hampering drug inclusion complexation. Release profiles of both drugs exhibited an initial fast release due to rapid dissolution of the complex located at or close to the NP surface followed by a delayed release phase due to drug diffusion and polymer swelling and degradation [104].

Subsequently, various other authors profitably exploited this combined approach to improve the effectiveness of CS NPs incorporating hydrophobic drugs, utilizing various kinds of non-ionic CDs and CS ionotropic gelation with TPP as the NP preparation method.

For example, Vyas et al. [105] developed CS NPs of the poorly soluble drug simvastatin loaded as a complex with HPβCD. Increased drug solubilization in the presence of increasing HPβCD amounts enabled a progressive increase in drug loading capacity into the NPs from the initial 2.9 or 3.6% (in the absence of CD) up to 7.9 or 8.2%, despite the reduction in the complex stability constant in the presence of CS, as found by phase solubility studies [105], in agreement with previous results [51]. As in the case by Maestrelli et al. [104], a biphasic release was found, with an initial burst effect followed by a delayed release phase [105].

The co-encapsulation into CS NPs of hydrophilic calcium folinate and hydrophobic methotrexate was successfully achieved by loading the first one as the plain drug and methotrexate as a complex with βCD or HPβCD [106]. The loading efficiency of methotrexate was higher in CS/βCD NPs than in CS/HPβCD NPs, due to the higher solubilizing and complexing ability of the native CD towards this drug. Release of both drugs presented a typical biphasic behavior, starting with a burst phase during the first hour and continuing with a slow release up to 24 h. The higher release rate of calcium folinate than methotrexate suggested a stronger sustained-release effect for the latter due to CD complexation [106].

The same authors developed NPs comprising O-carboxymethyl-CS or CS and βCD as oral delivery systems for the poorly soluble NSAID ibuprofen [107]. Both kinds of NPs exhibited significantly higher entrapment efficiency than the corresponding ones without βCD (80 and 93% vs. 50 and 45%, respectively). In vitro release studies showed that, at gastric pH, drug release from NPs made with the CS derivative was slower than from those with unmodified CS, while the opposite result was found at intestinal pH. The results indicated that NPs with the O-carboxymethyl-CS derivative were more suitable if a preferential drug release in intestinal medium is desired [107].

CD complexation and entrapment of the complex into CS NPs was exploited to enhance the poor solubility and poor permeability of two hydrophobic drugs, ranitidine.HCl and furosemide [108]. CS NPs were prepared by ionic gelation with TPP in aqueous solutions containing the complexes of the two drugs with HPβCD. The used procedure allowed to increase in solubility of both drugs, enabling effective entrapment into CS NPs (loading capacities of 18 and 14% for ranitidine and furosemide, respectively), and increased permeability across a Caco-2 cell monolayer, probably through electrostatic-induced interactions with paracellular proteins leading to the tight junction opening [108].

CS NPs loaded with warfarin as a βCD complex were prepared to improve transdermal delivery of the drug by joining the penetration-enhancing power of CS, the release-controlling abilities of CS NPs, and the solubilizing and stabilizing effects of βCD towards the hydrophobic drug warfarin, enabling its efficient encapsulation into hydrophilic NPs [109]. CS NPs loaded with the warfarin-βCD complex were prepared by ionic gelation with TPP, and the effects of varying the CS:TPP ratio and CS concentrations were investigated. High entrapment efficiency was obtained, reaching 94% at a 3:1 w/w CS:TPP ratio and 2 mg/mL CS concentration. Ex vivo permeation experiments through excised rat skin showed an almost linear increase in the amount of permeated drug according to zero-order kinetics, reaching about 60% after 8 h. The enhanced permeability of warfarin was ascribed to the synergistic effect of CS and βCD [109].

CS NPs containing plasmid DNA encoding interleukin-12 and βCD were prepared by crosslinking with TPP and characterized for physicochemical properties, loading efficiency, and cytotoxicity [110]. The NPs exhibited high DNA encapsulation efficiency (around 83%) and higher transfection ability compared to naked DNA in CT-26 colon carcinoma cells, without any cell toxicity, thus revealing as a promising effective carrier for gene delivery [110].

With the aim of developing an effective anticancer formulation of curcumin, whose therapeutic potential is strongly limited by its very low solubility, poor absorption, and fast metabolism, highly soluble curcumin-HPβCD hollow spheres were prepared by spray-drying and then incorporated into CS NPs [111]. The obtained curcumin-HPβCD-CS NPs exhibited superior in vitro release properties (around 70% drug released after 72 h vs. around 45% for NPs in the absence of CD and 8% for the drug alone) and the highest cytotoxicity (up to around 70%) determined by MTT assay in the SCC25 cell line. Oligo DNA loading into such NPs was also investigated, obtaining greater cellular delivery than analogous NPs without HPβCD. These results proved that CD not only increased curcumin solubility but also raised cellular uptake, suggesting the potential of the new formulation for dual hydrophobic drug–gene delivery [111].

In order to improve therapeutic efficacy and reduce serious side effects of paclitaxel, a scarcely soluble anticancer drug, CS NPs loaded with the drug as a complex with dimethyl-βCD (DMβCD) were prepared via ionic crosslinking with TPP [112]. The obtained NPs had positive zeta potential and good colloidal stability. Complexation with DMβCD dramatically increased paclitaxel water solubility, allowing a more than 30-fold increase in loading efficiency with respect to NPs loaded with the plain drug. As in previous cases illustrated above [104,105,106], the release profile of drug-DMβCD-loaded NPs presented an initial burst effect and a subsequent delayed release [112].

Complexation with CDs and incorporation into CS NPs were exploited to enhance the solubility of albendazole, a poorly soluble anticancer drug, and improve NP performance [113]. An optimized mixture of Tween 20 and HPβCD resulted in the highest increase in albendazole solubility, allowing loading of the therapeutic drug dose (100 µg/mL) [113].

Loading of poorly soluble anti-tuberculosis compounds isoniazid and isoconazole nitrate as complexes with βCD into CS-alginate MPs or NPs resulted in an enhancement of approximately 10 times of their antimycobacterial activity against *Mycobacterium tubercolosis* compared to the plain drugs [114].

The poor water solubility of the sulfa-antibiotic salazosulfapyridine was improved by complexation with dimethyl-βCD (DMβCD), and the complex was then incorporated into CS NPs, obtaining an effective sustained-release delivery system able to gradually release the drug over 24 h [115]. Entrapment efficiency of the CD-complexed drug into NPs was more than 90%, markedly higher than that of the plain drug (about 2%); moreover, the toxicity of NPs loaded with the complex was lower than that of the free drug [115].

The same authors [116] developed a sustained-release delivery system for mesalazine by complexing the drug with HPβCD to increase its water solubility and loading the complex into CS NPs. Entrapment efficiency and loading capacity increased with increasing amounts of inclusion complex, reaching up to 91.4% and 4.3%, respectively. The complex-loaded NPs typically showed an initial burst release and then a sustained-release phase over 24 h, while 95% of the drug was released by the complex as such after 2 h. Moreover, drug-HPβCD-loaded-NPs showed more marked anti-inflammatory activity than both free and CD-complexed mesalazine, as revealed by a more intense reduction in inflammatory mediators [116].

To reduce volatility, increase stability, and improve antimicrobial efficacy of *Cinnamomum zelanicum* essential oil, it was first complexed with βCD and then loaded into CS NPs [117]. The NPs loaded with the complex showed an entrapment efficiency about threefold higher than those loaded with the plain drug and reached an overall drug release of 71% vs. 49%, revealing it as a promising delivery system for essential oils [117].

To evaluate the effect of the combined use of two penetration enhancers on in vivo drug bioavailability of nasal formulations, MPs based on different w/w ratios of methyl-βCD (MβCD) and CS, and loaded with N6-cyclopentyladenosine, selected as model polar drug were obtained by spray-drying and compared with MPs containing a single enhancer (MβCD or CS) [118]. In vitro permeation studies through lipophilic membranes revealed that, when used alone, MβCD enhanced, while, on the contrary, CS reduced drug permeation; intermediate results, depending on their w/w ratios, were observed when using enhancers combinations. Moreover, in vivo studies after nasal administration to rats indicated that the presence of MβCD gave rise to enhanced drug concentration in the cerebrospinal fluid, and that optimal drug distribution between cerebrospinal fluid and blood circulation can be achieved by suitably modulating the content of the two penetration enhancers [118].

CD-containing CS NPs have been developed to improve the stability of the antigen ovalbumin and allow its sustained oral delivery [119]. Differently from the procedures followed in the cases illustrated above, CS NPs were prepared by a precipitation/coacervation method and then resuspended in a phosphate-buffered solution, where the previously prepared 1:10 mol:mol complexes of ovalbumin with βCD or carboxymethyl-hydroxypropyl-βCD (CM-HPβCD) or the free drug, were added; the solution was kept under stirring for 3 h at 25 °C. In vitro release studies evidenced a good retention of the drug into the NPs during the first 2 h at gastric pH for all formulations, followed by slow release at intestinal pH, which was more evident for the CD-containing-NPs. In vivo studies in mice revealed that NPs containing the drug as a complex with βCD or CM-HPβCD were about fourfold and about twofold more effective than ovalbumin solution and free ovalbumin-loaded CS NPs, respectively, in inducing intestinal mucosa immune response, indicative of a synergistic effect between CD and CS [119].

Clarithromycin nasal mucoadhesive MPs intended for use in respiratory tract infections were realized by complexing the drug with βCD to adequately increase its solubility and then entrapping the complex into NPs of CS selected as a mucoadhesive agent [120]. MPs were prepared by an emulsion–lyophilization technique. Entrapment efficiency increased with increasing βCD concentration. The best formulation, optimized by a 2^4^-factorial design, reached an entrapment efficiency of about 74%, mucoadhesion on excised sheep nasal mucosa of about 81%, and a maximum drug amount permeated in vitro of 80%, and was stable for at least six months [120].

A delivery system of tetrahydrocurcumin based on its complexation with βCD to improve its poor solubility and the entrapment of the complex into CS particles, to exploit the carrier properties of this polymer, has been realized, aimed to deliver the drug into cancer cells and produce a cytotoxic effect [121].

CS-βCD NPs co-loaded with curcumin and vitamin C have been prepared to improve the chemical stability and low bioavailability that restrict their effectiveness as bioactive functional factors and to obtain independent co-delivery [122]. Co-encapsulation resulted in an increase in stability of both compounds and enhanced their antioxidant abilities with respect to the single ingredients. Release profiles of both compounds were pH-dependent and showed an initial burst effect followed by prolonged release over 12 h [122].

Inclusion complexes of the antibiotic drug metronidazole with βCD and HPβCD have been prepared to increase its solubility and stability. The metronidazole-HPβCD complex not only showed the best dissolution properties but also exhibited a more marked increase in antibacterial activity and was therefore selected for incorporation into CS NPs, which allowed prolonged and controlled release of the drug, reaching almost 100% release after 6 h [123].

#### 6.5.2. Drug-in Anionic CD-in CS MPs and NPs

Other authors applied the same CD/CS combined strategy but utilized negatively charged CDs, in particular sulfobutylether-βCD (SBEβCD) and carboxymethyl-βCD (CMβCD), to exploit not only their complexing and solubilizing abilities towards drugs, but also their capacity to form ionic intermolecular interactions with the positively charged amino groups of CS.

A new type of NPs based on CS and CMβCD was developed by Krauland and Alonso [124], and its potential as a delivery system for macromolecular drugs was investigated. NPs were prepared by CS ionotropic gelation with the anionic CD, either alone or in the presence of TPP. The resulting NPs were stable at pH 6.8 and 37 °C (simulating intestinal conditions) for at least 4 h and allowed high loading of both insulin and heparin (selected as macromolecular model drugs), with association efficiencies ranging from 85 to 93% and from 69 to 71%, respectively. Release profiles strongly depended on the molecule type, presenting very rapid release in the case of insulin and slow, prolonged release for heparin, due to its stronger association with the NPs [124].

This same strategy was used to develop NPs consisting of CS and SBEβCD or CMβCD intended for nasal administration for systemic release of macromolecules such as insulin [125]. CS/CD NPs proved to be able to reversibly decrease the transepithelial resistance of a Calu-3 cell monolayer by opening tight junctions, thus increasing membrane permeability, and to overcome the nasal mucosal barrier, as proved by in vivo experiments in rats. CS/CD NPs loaded with insulin showed high association efficiency (>88%) and, when nasally administered to rabbits, reduced more than 35% the blood glucose levels [125].

The effectiveness of a non-ionic CD, namely HPβCD, and two anionic ones, i.e., SBEβCD and CMβCD, characterized by different charge densities (0.02958 and 0.02181 mol/g, respectively) in forming CD-containing CS NPs was investigated and compared [126]. NPs containing HPβCD were prepared by classic ionotropic gelation with TPP, with the CD dissolved in either the CS or TPP solution. NPs containing anionic CDs were prepared using the same ionotropic gelation, either without or with TPP. Incorporation efficiency of anionic CDs within the NPs increased with increasing CD amount in the starting solution and was higher for SBEβCD than for CMβCD, and much higher than that of HPβCD. However, CS incorporation efficiency significantly enhanced in the presence of HPβCD, suggesting the ability of the non-ionic CD to interact with the cationic polymer via hydrophobic forces and/or hydrogen bonds [126]. Moreover, all CD-containing CS NPs (and particularly those with HPβCD) showed higher stability in simulated intestinal medium than control NPs without CD, proving their superior performance as drug carriers [126].

Considering the known ability of CDs in improving chemical stability of peptides and proteins, an oral delivery system for the peptide drug glutathione was developed based on CS NPs as drug carriers and simultaneously exploiting the stabilizing and modulating-release properties of SBEβCD [127]. NPs containing or not the anionic CD were prepared by ionic gelation, in the absence or presence of TPP, respectively. Encapsulation efficiency of the peptide in the presence of SBEβCD was around 25%. X-ray photoelectron spectroscopy enabled quantitative determination of glutathione in-depth distribution in the inner core of the NP, showing that CS acts as a protective coating for the drug [127].

Further studies confirmed that CS/CD NPs are more effective carriers for oral peptide delivery than simple CS NPs [128]. In fact, the presence of SBEβCD allowed an about 3.5 times increase in entrapment efficiency of the model peptide glutathione; moreover, transport studies showed that CS/SBEβCD promoted a similar increase in drug permeability as CS NPs in the proximal intestine segment, but a significantly higher increase in the distal segment [128].

The potential of nanocarriers composed of CS and anionic CDs (SBEβCD or CMβCD) as non-viral gene delivery system was investigated by evaluating their ability to enter epithelial cells and promote gene expression in a Calu-3 cell culture model [129]. NPs spontaneously formed by ionotropic gelation by mixing the solution containing CS (at low- or medium-Mw) with that containing the anionic CD, TPP, and a plasmid DNA model encoding secreted alkaline phosphatase. The obtained NPs showed high DNA association efficiency (>90%) and were effectively internalized by cells. Transfection efficiency, determined by the amount of secreted gene product, proved that all CS/CD formulations elicited a better response than naked DNA (control), with the best values obtained for NPs composed of low-Mw CS [129].

The development of CS NPs using SBEβCD as a polyanionic CS crosslinker and as a complexing agent for econazole nitrate was evaluated as a potential approach for ocular delivery of this poorly soluble antifungal drug [130]. Drug content in the NPs varied from 13 to 46% and increased with increasing concentrations of CS and SBEβCD. The obtained CS/SBEβCD NPs showed controlled and prolonged drug release up to 8 h and proved better antifungal activity at the ocular level than the simple drug solution in in vivo studies in albino rabbits [130].

NPs made of CS and CMβCD were investigated for encapsulation of the NSAID sulindac [131]. NPs were prepared by ionotropic gelation by dropwise addition of solutions containing increasing amounts of the sulindac-CMβCD complex to the CS solution, exploiting ionic interactions between negatively charged CD and positively charged CS and avoiding the use of TPP. Drug entrapment efficiency increased from 48 to 85% with increasing CMβCD amount. Release profiles indicated prolonged release over 24 h, although a rapid release phase was observed during the first 3 h [131].

A similar approach, consisting of the preparation of CS MPs or NPs using SBEβCD as both a crosslinking agent for CS and a solubilizing agent for the hydrophobic drug hydrocortisone, was utilized by Fülop et al. [132]. The authors proved that both particle size and drug release rate of MPs and NPs can be modulated by suitably varying the starting concentrations of CS and CD. Entrapment efficiency of the MPs and NPs decreased with increasing CS concentration during their preparation [132].

This same strategy was exploited to obtain an effective encapsulation into CS NPs of a tea polyphenol loaded as a complex with SBEβCD; as expected, entrapment efficiency increased with increasing SBEβCD amount, reaching up to 93% [133].

With the same modality, CS NPs loaded with naringenin as an SBEβCD complex to improve its solubility were prepared and investigated for topical ocular delivery [134]. Incorporation of the complex into CS NPs allowed a more sustained release than the complex. In vivo studies on rabbits showed that drug- SBEβCD-loaded-CS NPs did not cause any irritating effects and gave rise to an about 4.6 times increase in the AUC (ng.g/mL) in the rabbit aqueous humor compared to the simple drug suspension [134].

Analogously, more recently, CS/SBEβCD NPs were developed to improve ocular delivery of levofloxacin [135]. Use of SBEβCD enabled significantly higher entrapment efficiency compared to NPs prepared without CD using TPP as a crosslinking agent (48 vs. 25%), as well as more sustained release with a reduced burst effect (20% vs. 60% drug released after 1 h). The high positive zeta potential of the NPs promoted interaction with ocular tissues, prolonging in situ residence time. The obtained NPs exhibited twofold higher antibacterial activity against Gram-positive and Gram-negative bacteria than the plain drug [135].

In the same way, CS/SBEβCD NPs coated with thiolated, low-molecular-weight hyaluronic acid have been realized as carriers to enhance the ocular bioavailability of indomethacin by increasing drug solubility and prolonging in situ residence time in the conjunctival sac [136]. The obtained coated NPs presented no irritancy/toxicity effects and exhibited higher mucoadhesive properties than uncoated ones; nevertheless, uncoated NPs exhibited greater in vitro permeation ability, probably due to their smaller dimensions, and could be therefore useful for treating posterior segment disorders [136].

An ophthalmic delivery system consisting of mucoadhesive NPs of dexamethasone was formulated to improve drug bioavailability and therapeutic efficacy in anti-inflammatory therapy [137]. The drug was complexed with SBEβCD to improve its aqueous solubility and then incorporated into CS NPs formed by ionotropic gelation, by virtue of the interactions between cationic CS and anionic CD. The obtained NPs showed good entrapment efficiency (87%), owing to increased solubility of the complexed drug, as well as a prolonged in situ residence time and enhanced drug permeation through excised bovine corneal membrane, owing to the mucoadhesive and permeation-enhancing properties of CS [137].

To develop an intranasal delivery system aimed at nose-to-brain delivery of dopamine, the drug was complexed with SBEβCD to improve its stability against light degradation and then entrapped into NPs of glycol-CS (a more soluble derivative of CS) formed by exploiting interactions between the anionic CD and the cationic CS derivative [138]. A more controlled release and better stabilizing effect was found compared to glycol-CS NPs loaded with free drug using TPP as a crosslinker. Repeated intranasal administrations of the dopamine-SBEβCD-glycol-CS NPs into the right nostril of rats gave rise to a significant enhancement of neurotransmitter levels in the ipsilateral striatum; moreover, administration of these fluorescently labeled NPs allowed detection of NPs only in the right olfactory bulb, without any tissue injury, proving their potential as carriers for dopamine nose-to-brain delivery [138].

Drug-in CD-in CS NPs were developed as potential carriers for idebenone nose-to-brain targeted delivery [139]. SBEβCD was used as a complexing agent for idebenone and as a crosslinking agent for CS, allowing production of NPs in aqueous solution with high entrapment efficiency of the poorly soluble drug. Overloaded NPs were also prepared by dissolving an additional amount of the idebenone-HPβCD complex into the aqueous solution of CS or into the solution of idebenone-SBEβCD complex before NP formation. Higher entrapment efficiency was obtained for overloaded NPs (72.7% vs. 42.1%). Non-overloaded NPs showed a slow, prolonged release without a burst effect, whereas an evident burst effect and a higher release rate were observed for overloaded NPs, whose behavior depended on the addition of the soluble complex to the CS or the SBEβCD solution. The permeation ability through excised bovine nasal mucosa followed the order: plain drug < non-overloaded NPs < overloaded NPs < drug-HPβCD complex [139], thus confirming the previously observed ability of HPβCD to increase idebenone permeation [140].

CS/SBEβCD/TPP NPs loaded with the hydrophobic flavonoid quercetin have been developed and evaluated for their ability to arrest Quorum Sensing (QS) in bacteria in comparison with analogous NPs not containing CD [141]. Presence of SBEβCD allowed up to an eightfold increase in drug loading efficiency and obtained prolonged release over 6 h, while 90% of drug was released within 1 h from NPs loaded with free quercetin. Bioassays against an *Escherichia coli* Top 10 QS biosensor showed that CS NPs loaded with the quercetin-SBEβCD complex had a stronger anti-QS action than the unloaded ones, indicating a synergistic effect among CS, CD, and flavonoid; moreover, the highest anti-QS effects were observed for formulations with the highest SBEβCD content and, consequently, the highest content of the associated quercetin [141]. The same authors performed an analogous study evaluating the QS-arresting efficacy in bacteria of the flavonoid naringenin loaded into NPs coated with CS and surface-adsorbed with SBEβCD; results indicated that incorporation of the drug in such multifunctional NPs increased its activity in quenching the QS response against an *Escherichia coli* Top 10 biosensor and decreased cytotoxicity [142].

A nano-formulation for delivery of ibrutinib (a tyrosine-kinase-inhibitor antitumoral drug), has been realized by loading the drug into CS/SBEβCD NPs formed by the ionotropic gelation method, using CS and SBEβCD as the cationic and anionic phases, respectively [143]. Both loading capacity and entrapment efficiency of the hydrophobic drug increased with increasing SBEβCD content, reaching maximum values of about 13 and 77%, respectively. The authors proved that the obtained NPs showed mucoadhesive properties and maintained drug activity on the target kinase. In vitro release studies indicated that while the free drug suspension showed very high release rate at gastric pH and very poor release at intestinal pH due to pH-dependent solubility, the CS/SBEβCD NPs presented a pH-independent sustained-release profile, slower than that of the ibrutinib-SBEβCD solution [143].

Cinnamaldehyde-SBEβCD-loaded CS hydrochloride (CSH) NPs have been prepared via ionic gelation by dropwise addition of an aqueous solution of the inclusion complex with the anionic CD to that of the cationic polymer CSH [144]. High entrapment efficiency (86.34%) was achieved thanks to drug complexation with SBEβCD. The obtained NPs showed improved drug stability against oxidation and volatility and exhibited very satisfying slow-release properties and better antibacterial activity against *Escherichia coli* and *Streptococcus aureus* than the plain drug [144].

A novel delivery system for rifampicin was proposed based on drug complexation with SBEβCD and subsequent incorporation of the complex into CS NPs [145]. NPs were prepared by ionic gelation exploiting interactions between CS and SBEβCD, eventually also adding other crosslinkers such as carboxymethylcellulose (CMC) or TPP. CS/SBEβCD NPs showed the highest drug loading (7.7% vs. 4.8 and 3.5% for CS/SBEβCD/CMC and CS/SBEβCD/TPP NPs, respectively), the highest positive zeta potential, the best stability, and the strongest mucoadhesive properties. Both CS/SBEβCD and CS/SBEβCD/CMC NPs showed good sustained-release profiles and enhanced antimicrobial effect compared to free rifampicin, with the former being more effective against *Streptococcus aureus* and the latter against *Escherichia coli* [145].

Doxorubicin-loaded NPs, prepared by ionic gelation using cationic CS or its derivative 2-hydroxypropyl-trimethylammonium chloride-CS bearing folic acid (CSF) and anionic CMβCD were developed [146]. NPs showed good entrapment efficiency (73 and 75%, respectively), higher than that of CS NPs without CD previously reported [147], and exhibited reduced cytotoxicity and enhanced antitumor activity than the plain drug, while providing controlled and prolonged drug release. Moreover, CSF/CMβCD NPs showed the highest antioxidant activity due to the presence of folic acid [146].

The hypoglycemic activity of hesperidin, whose oral bioavailability is limited by its poor solubility, was improved by complexing the drug with SBEβCD and then loading the complex into CS NPs formed by ionotropic gelation [148]. The best NP formulation reached 77.5% entrapment efficiency, showed sustained release over 12 h, and exhibited a significantly stronger hypoglycemic effect in rats not only with respect to the plain drug but also to the complexed drug, showing a synergistic effect between CS and SBEβCD in improving drug bioavailability. This synergistic effect was attributed by the authors to improved drug solubility (due to CD complexation) and to the ability of SBEβCD to reduce mucus layer viscosity, with consequent increases in intestinal permeability, associated with the mucoadhesive properties and enhanced paracellular pathways provided by CS [148].

A novel mucoadhesive in situ gel system containing quercetin-SBEβCD-loaded CS NPs was realized for the treatment of vulvovaginitis [149]. The complex-loaded CS NPs prepared by ionic gelation exhibited higher antibacterial activity against both *Streptococcus aureus* and *Pseudomonas Aeruginosa* than the simple drug solution. Incorporation of NPs into an in situ gel based on a poloxamer-CS combination increased residence time at the vaginal level, providing prolonged release and good antimicrobial action [149].

## 7. Delivery Systems Based on Purposely Synthesized CS-CD Composites

CD-linked-CS derivatives have been synthesized with the aim of joining the several favorable properties of CS with the CD complexing/solubilizing ability, thus overcoming the poor affinity of CS itself towards hydrophobic molecules and creating biocompatible, biodegradable carriers with high loading efficiency towards a wider range of drugs.

Different synthetic strategies can be used for CD grafting onto CS and CS derivatives [150], and the resulting compounds generally present the cumulative abilities of the starting components [151].

The obtained new products, after careful characterization proving the actual maintenance of biocompatibility, biodegradability, and absence of cytotoxicity of the starting components, as well as of their other beneficial properties, including, respectively, the CS mucoadhesive power and the CD inclusion complexation ability, can be used as fruitful excipients for a variety of drug delivery systems intended for different administration routes.

For example, Venter et al. [152] synthesized a βCD-grafted-CS polymer that maintained a good mucoadhesive strength, only slightly weaker (about 13%) compared to the parent CS, and showed satisfying inclusion properties towards the model guest molecule (+)-catechin, although about fivefold lower than the parent βCD. The reduced affinity of the guest molecule for the grafted βCD has been attributed to a less favorable entropy change probably related to the covalent binding to the CS polymer [152].

In another study, in order to favor CD mobility and limit possible steric hindrance that could limit its complexing ability, βCD was grafted onto CS using a citrate spacer; the obtained conjugate was then quaternized with glycidyl trimethylammonium chloride groups to enhance its aqueous solubility [153]. In vitro mucoadhesive properties of the final compound were found to be dependent on the degree of quaternization and evidenced a positive role of the citrate spacer; cytotoxicity studies pointed out reduced cytotoxicity against buccal mucosal cells for the quaternized CD-CS conjugate compared to simple quaternized CS [153].

However, it has been considered that final quaternization of the CD-CS conjugate could lead to a reduction in the CD complexing capacity. Therefore, in a following study, in order to prevent any potential steric hindrance effects, Piras et al. [154] first synthesized a water-soluble quaternary-ammonium-CS derivative and then functionalized it by grafting highly soluble methyl-βCD (MβCD) onto the polymer through a 10-atom-long spacer. The obtained product was able to effectively complex dexamethasone, selected as a model drug, and maintained good mucoadhesive power and safety, indicating its suitability as a solubilizing agent for ophthalmic formulations [154].

Similarly, the same research group synthesized a water-soluble CS derivative by reaction with 2-chloro-N,N-diethylethylamine, and then MβCD was covalently linked to the quaternized CS using 1,6-hexamethylene diisocyanate as a spacer [155]. NMR spectroscopic studies evidenced deep inclusion of the model drug prednisolone phosphate into the MβCD cavity and pointed out a synergistic effect with the polymer backbone in interactions with the drug, proving the superior affinity for prednisolone of the CD-grafted-quaternized CS than the parent quaternized CS [155].

Considering the known antimicrobial effect of CS, as well its absorption-enhancing power, and the solubilizing ability of CDs by inclusion complexation, a new polymer was synthesized by grafting mono-6-deoxy-6-(p-toluenesulfonyl)-βCD onto CS (CD-g-CS) and tested for antimicrobial activity [156]. Agar diffusion bioassays proved antimicrobial activity of the obtained CD-g-CS polymer against *Escherichia coli* and *Staphylococcus xylosus*, which was ascribed to bacterial membrane disruption and cell death. Furthermore, the authors demonstrated that CD-g-CS was able to increase solubility of the antibiotic drug doxorubicin and promote its uptake by *Staphylococcus xylosus*, joining the properties of CD and CS [156].

The strategy of using purposely synthesized CS-CD composites has also been applied to the development of advanced hydrogel formulations. For instance, different CD-grafted CS hydrogels were obtained starting from carboxymethyl-CS (CMCS) and carboxymethyl-βCD (CMβCD) utilizing a hydro-soluble carbodiimide as a crosslinker [157]. The resulting CMβCD-g-CMCS hydrogels displayed clearly superior absorption ability towards acetylsalicylic acid compared to CMCS hydrogel; this better performance was directly related to CD content within the hydrogel, indicating that most drug molecules were actually entrapped in the CD cavity. Moreover, CMβCD-g-CMCS hydrogels provided slower and more controlled release of the drug compared to CMCS hydrogel [157].

Zhang et al. [158] prepared a novel supramolecular hydrogel by a Diels–Alder reaction in aqueous media between furfural-functionalized CS and functionalized HPβCD. The authors proved that the introduction of CD into the CS hydrogel improved thermal stability, increased drug adsorption ability, and allowed controlled drug release over 24 h [158]

A series of pH-responsive hydrogels was produced for controlled release of acyclovir using different w/w ratios of βCD, CS, methacrylic acid, and N’N’-methylenebis-acrylamide. The obtained hydrogels showed higher thermal stability than their single components and high loading efficiency (ranged between 75 and 90%) [159]. Drug release studies in simulated gastrointestinal media showed slow drug release at gastric pH and higher release rate at intestinal pH, according to the swelling behavior of the hydrogels [159].

In the field of wound-dressing formulations, a biocompatible sponge material was obtained by addition of βCD to CS solutions, using dialdehyde starch as a crosslinker, followed by lyophilization [160]. This sponge material can be prepared in the presence of active compounds, such as the natural alkaloid berberine, allowing high drug loading into the matrix, due to drug complexation with βCD, as well as adsorption on the surface of the highly porous sponge structure. Thus, the obtained material can be used for preparing sponge topical delivery systems for sustained release of berberine [160].

CD-grafted-CS polymers have also been used for the development of new kinds of nanoparticles (NPs) as more effective carriers of poorly soluble drugs.

For instance, NPs based on a βCD-grafted-CS (CD-g-CS) polymer have been evaluated as a delivery system for improving the solubility and controlling the release of the poorly soluble NSAID ketoprofen [161]. The CD-g-CS polymer was synthesized from mono-6-deoxy-6-(p-toluenesulfonyl)-βCD and CS, and ketoprofen-loaded CD-g-CS NPs were prepared via the ionic gelation method by TPP addition. CD-g-CS NPs exhibited greater stability in phosphate-buffered saline with respect to the corresponding CS NPs and also higher drug entrapment efficiency (75.3 vs. 55.4%). All the tested formulations showed a pH-responsive controlled release, with an initial burst effect followed by prolonged release up to 12 h [161].

Other authors exploited the same strategy to realize NPs consisting of βCD grafted onto N-maleoyl-CS polymer (used as a soluble CS derivative) (CD-g-NMCS), as a potential carrier for the drug delivery of poorly soluble drugs compared with CS NPs [162]. Ketoprofen was selected as the model drug, and drug-loaded NPs were prepared according to the ionic gelation method by TPP addition. CD-g-NMCS NPs showed higher entrapment efficiency than CS NPs (65.4 vs. 55.4%), better stability in simulated biological fluids (up to 3 days vs. only 24 h), attributed to the more compact structure of the βCD-grafted polymer, and more sustained release, with a reduced initial burst effect. Furthermore, after intravenous administration to rats, CD-g-NMCS NPs showed a longer half-live (686 vs. 486 min) and a significant increase in AUC_0–24h_ (2632 vs. 1894 µg.min/mL) and mean residence time (816 vs. 531 min) compared with CS NPs, indicative of more prolonged release. These results were attributed to the presence of CD, which complexed the drug, improving its solubility and slowing down its release [162].

In other studies, carboxymethyl-βCD (CMβCD) was covalently grafted onto CS by an amidation reaction mediated by EDC/NHS (1-ethyl-3-(3-dimethlaminopropyl) carbodiimide hydrochloride/N-hydroxysuccinimide) [163,164]. The obtained polymer (CMβCD-g-CS), joining the inclusion complexation ability of CD with the mucoadhesive and penetration-enhancing properties of CS, was used to obtain NPs as carriers for oral protein delivery; they were prepared by ionic gelation with TPP, in the presence of bovine serum albumin [163] or insulin [164], respectively. CMβCD-g-CS NPs reached an optimal entrapment efficiency, around 77% for albumin and 57% for insulin, at protein/carrier ratios of 5% and 10%, respectively, and enabled pH-dependent controlled release of the protein over 8–12 h. The insulin-CMβCD-g-CS NPs were able to promote protein internalization into Caco-2 cells and induce reversible opening of tight junctions between cells; oral administration of insulin-CMβCD-g-CS NPs to diabetic mice gave rise to a 51% decrease in initial blood glucose levels, while no effect was observed after oral administration of an insulin solution [164].

A new targeted ligand polymer was synthesized by grafting folate and carboxymethyl-βCD (CMβCD) onto trimethylCS (FC-TCS), with the potential to act as a carrier of antitumor and genetic drugs. The polymer was used to prepare NPs (via ionotropic gelation, using TPP as a crosslinker), which were evaluated as co-carriers of doxorubicin and siRNA [165]. Optimal entrapment efficiency values (85.73% for doxorubicin and 95.66% for siRNA) was achieved at a 5% ratio of doxorubicin (or siRNA)/carrier ratio. The NPs effectively protected siRNA from decomposition by nuclease in serum. Furthermore, both drugs showed pH-dependent prolonged release, and cytotoxicity studies indicated a significant improvement in antitumor action of the co-loaded NPs, due to a synergistic effect between doxorubicin and siRNA [165].

A different strategy was proposed by Chen et al. [166], who realized silica-coated Fe_3_O_4_ magnetic NPs functionalized with epoxy groups, then grafted with βCD and finally coated with CS, aimed at targeted delivery of hydrophobic drugs such as ibuprofen. The CD-CS double-layered coating enabled us to obtain biocompatible magnetic NPs with good stability and high drug loading capacity (82.87 mg/g), owing to their positive zeta potential and the drug inclusion ability provided by CS and βCD, respectively. The release profile presented an initial fast release followed by a sustained-release phase, which could be affected by pH, temperature, and magnetic field [166].

Le Deygen et al. [167] recently synthesized conjugates of CS with two βCD-derivatives, i.e., O-p-toluenesulfonyl-mono-(6-(hexamethylenediamine)-6-deoxy)-βCD (TsNH2-βCD) and O-p-toluenesulfonyl-HPβCD (Ts-HPβCD), as mucoadhesive carriers intended for the prolonged delivery of levofloxacin. Complexation of the drug with such conjugates allowed an about fourfold slower release with respect to the complex with plain CDs and about twentyfold slower than the free drug, while maintaining unchanged antibacterial activity [167].

A novel insoluble polymer, synthesized by grafting the three native CDs, namely αCD, βCD, and γCD, onto CS, was evaluated for its ability to selectively adsorb and remove pharmaceutical contaminants present in aqueous solutions [168]. The obtained polymer presented a porous structure with high swelling ability and showed excellent adsorption properties towards ibuprofen and progesterone, selected as model pharmaceutical pollutants, reaching up to 75 and 90%, respectively. The adsorption mechanism was guided by both inclusion complex formation and electrostatic interactions with the polymer. The proposed approach appears to be a selective and advantageous method for pollution remediation [168].

## 8. Conclusions

A comprehensive overview of the main approaches explored for developing new, more effective drug delivery systems based on the combined use of CDs and CS has been provided. It has been displayed how such a dual strategy can overcome the issues of these materials when utilized individually and join their respective advantages into a single delivery system.

Numerous examples of applications have been illustrated. They range from solid oral dosage forms to hydrogels, film formulations, and micro- and nanoparticles intended for different administration routes and carrying different kinds of drugs, such as especially NSAIDs, antimicrobial and antitumoral agents, macromolecules such as peptides or proteins, or genetic materials.

For each typology of delivery system or administration route considered, the main drawbacks and limitations, as well as the various possible benefits that can be achieved by the dual CD/CS approach, have been described.

Especially attractive are the results obtained with nasal formulations for systemic or nose-to brain drug delivery, which exploit, at the same time, the solubilizing ability of CDs to enhance the loading of hydrophobic drug, the mucoadhesive power of CS to prolong in situ residence time and obtain prolonged release, and the synergistic effect of CS and CD in promoting drug permeation [93,94,95,96,97,118,120,125,138,139].

Of particular interest are also the results obtained in antitumoral therapy, where the simultaneous presence of CD and CS enabled enhancement of drug loading, improvement and prolongation of its release, and enhancement of its antitumor activity, thus allowing a reduction in dosage and adverse side effects [111,112,146,165].

In conclusion, based on the several and very significant benefits that can be attained, in terms of enhanced delivery system characteristics and increased drug therapeutic efficacy of drugs, as well documented in the literature, it is definitely worthy of interest to further investigate the effects of the concomitant use of CDs and CS to overcome the issues/limitations encountered and find increasingly efficient new applications.

In particular, a very important matter deserving more in-depth investigation is how to take advantage of the combined use of CD-CS to obtain suitable site-specific delivery systems able to effectively and selectively carry and release drugs to target cells (particularly in cancer therapy), thereby maximizing therapeutic efficacy. In this regard, important advancements could be achieved by developing suitable CD and/or CS derivatives and/or functionalizing these polymers with specific ligands. However, it must be considered that this approach leads to a series of challenges, starting from the synthesis of such derivatives to their careful characterization and health approval as safe materials for clinical use, thus strongly delaying the prospects for their actual practical applications.

## Data Availability

No new data were created or analyzed in this study.
